# New Type of Asphalt Concrete with Bitumen Emulsion in Terms of Type and Quantity of Binder

**DOI:** 10.3390/ma18235437

**Published:** 2025-12-02

**Authors:** Maciej Krasowski, Przemysław Buczyński, Grzegorz Mazurek, Matúš Kozel

**Affiliations:** 1 Department of Transportation Engineering, Faculty of Civil Engineering and Architecture, Kielce University of Technology, Al. Tysiąclecia Państwa Polskiego 7, 25-314 Kielce, Poland; p.buczynski@tu.kielce.pl (P.B.); gmazurek@tu.kielce.pl (G.M.); 2 Department of Technology and Construction Management, Faculty of Civil Engineering, University of Žilina, 8215/1, 01026 Zilina, Slovakia; matus.kozel@uniza.sk

**Keywords:** cold mix asphalt, polymer powder, design of experiment, bitumen emulsion

## Abstract

This paper presents the effect of modifiers on the properties of a mixture of asphalt concrete with bitumen emulsion (ACBE). The mineral-asphalt mixture is the only one that can be produced using the cold-mix technology (CMA). The theoretical part of the article details the characteristics of the methods for producing mineral-asphalt mixtures in terms of their production temperature. Thus, hot (HMA), half-warm (H-WMA), warm (WMA) and cold (CMA) mixtures are discussed. The research section presents the design of the asphalt concrete composition with bitumen emulsion, the research methods, the experiment design and the research results. The design of the mixture of asphalt concrete with bitumen emulsion was carried out in accordance with the guidelines set out in EN 13108-31. In the experiment, Portland cement (C), bitumen emulsion (A), synthetic latex (styrene-butadiene rubber SBR) (B) and redispersible polymer powder EVA (polyethylene-co-vinyl acetate) (P) were used as modifiers. Twenty-four mixtures were designed as part of the experiment, according to the 3^4^ experiment design. The following physical and mechanical properties were assessed in the design of the research: air void content V_m_, water ab-sorption n_w_, indirect tensile strength ITS and IT-CY stiffness modulus. When analysing the research results, the authors observed a noticeable impact of the content of asphalt (A) and synthetic latex (B) on the air void content V_m_. A significant effect was also observed for the interaction of Portland cement (C) and redispersible polymer powder (P) on the indirect tensile strength ITS. The next step was the optimisation of the ACBE mixture composition, which effect made it possible to identify the optimum amounts of modifiers in the mixture of asphalt concrete with bitumen emulsion (ACBE), which constituted recommendations for the requirements for mixtures of asphalt concrete with bitumen emulsion.

## 1. Introduction

Pavements made from mineral-asphalt mixtures have been used successfully throughout the world for many years. Over the years, production processes have been refined through various modifications [1]. A major step in the development of asphalt production methods was the commencement of modifications using natural and synthetic polymers. In the early 1980s, a styrene-butadiene-styrene (SBS-type) polymer was produced for the first time [2]. In Europe and the USA, the use of polymer-modified bitumens began in the second half of the 1980s [2]. Polymer-modified bitumens appeared in Poland in the 1990s [3].

Over the years, mixture production methods and technologies have evolved as well. The oldest and most common technology is the production of hot-mix asphalt (HMA) [4]. This technology involves heating the mineral aggregate and asphalt separately. The aim is to reduce the viscosity of the asphalt (liquefaction), and heating the aggregate helps to coat the aggregate grains with the binder [4]. Adhesive agents are added to facilitate and improve adhesion. The production temperature of the mixture is between 150 °C and 190 °C [4]. Due to high production temperatures, it is believed that HMA mixtures are not beneficial for the environment [5]. This is associated with significant atmospheric emissions of gases. HMA production is also very energy-intensive [6,7].

A more environmentally friendly mixture with a lower production temperature than in the case of HMA is the “warm” mix asphalt. This technology has been developed since the early 1990s, mainly in the USA [8]. Eventually, it emerged in Europe. This method involves reducing the viscosity of the asphalt without heating it to high temperatures, like HMA [9]. One way of performing this is to use suitable additives such as waxes or fatty acid amides. These chemicals are usually dosed at a rate of 0.1–3% of asphalt weight in the mixture. Another way to lower the viscosity of asphalt is to use the foamed bitumen technology [10]. HMA technology involves adding water and pressurised air to the hot asphalt. The hot asphalt (at approximately 160–180 °C) is poured into a special system where water and compressed air can be added. Then, a small amount of water is injected into the hot asphalt line. As a result, the water rapidly evaporates, forming tiny bubbles surrounded by a thin bitumen film. The resulting foamed bitumen is mixed with mineral aggregate [11]. The production temperature of the WMA mix is approximately 100–150 °C [12,13].

Another type of mineral-asphalt mixture is the “half-warm” mix asphalt (H-WMA). This technology was invented in 1999 and is partly classified as a WMA mix. That is due to the bitumen foaming technology involved [14]. Using a suitable method of bitumen foaming, it is possible to pour mixtures at a temperature of 60–100 °C. An example of the technology used is Evotherm ET [15]. The method involves heating the mineral aggregate to 120–130 °C and then adding a bitumen emulsion with the polymer modifier SBR (styrene–butadiene–rubber). The added ingredients initiate the bitumen foaming process [16]. The final mixture has a temperature of 90–100 °C [17,18].

**Figure 1:**
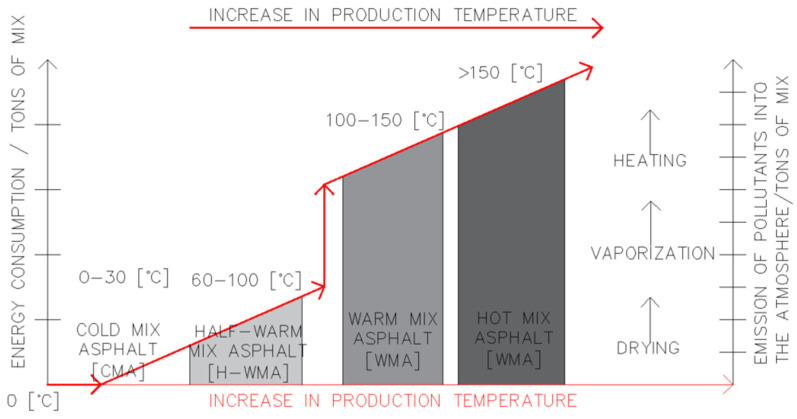
A breakdown of mineral-asphalt mixtures according to the temperature of production [25].

The last of the technologies is the cold mix asphalt (CMA). This method involves producing mixtures at temperatures in the 0–30 °C range. This is possible by using bitumen emulsion as a binder [19]. The bitumen emulsion is added to the wet mineral mix without heating. Upon contact between the aggregate and the emulsion, the emulsion breaks down. As a result of compaction, water is removed from the mixture, and the dispersed bitumen forms bonds between the grains of the mineral material [20,21]. This method is successfully used worldwide [22,23,24]. The following figure (Figure 1) shows a breakdown of mineral-asphalt mixtures according to the temperature of production.

The CMA method has many advantages. An example is the low production temperature, which translates into lower energy requirements, resulting in lower emissions of harmful gases. Additionally, bitumen emulsions may contain additives (e.g., reinforcing fibres) [26] that remain after water evaporation, yielding composite asphalt with improved mechanical properties. What is more, the low production temperature translates into longer times available for the transport and pouring of the mixture. It is also possible to commission the completed layer more quickly than for the conventional HMA mixture. Despite its many advantages, the mix also has disadvantages. The main disadvantage cited by researchers is the high air void content, Vm, and low frost and water damage resistance [27,28].

Consequently, it seems necessary to look for methods to improve the CMA technology. Current global trends aimed at reducing environmental pollution [29] are forcing the use of environmentally friendly materials that generate low atmospheric emissions. The CMA mix seems to be the perfect starting point for further development. The introduction of modifiers may prove to be a way of improving some of the properties for which the conventional CMA technology is not chosen by contracting authorities and contractors. It is necessary to look for the optimum amount of binders to produce a new type of asphalt concrete with bitumen emulsion.

The subject of the research is the mineral-asphalt mixture produced using the CMA technology. The authors have selected the mixture of asphalt concrete with bitumen emulsion (ACBE) for the analysis. According to the definition [30], an ACBE mix is a mix in which a continuous mineral skeleton forms an interlocking structure in which all or part of the binder is added in the form of bitumen emulsion.

## 2. Materials and Methods

### 2.1. Materials

**Figure 2:**
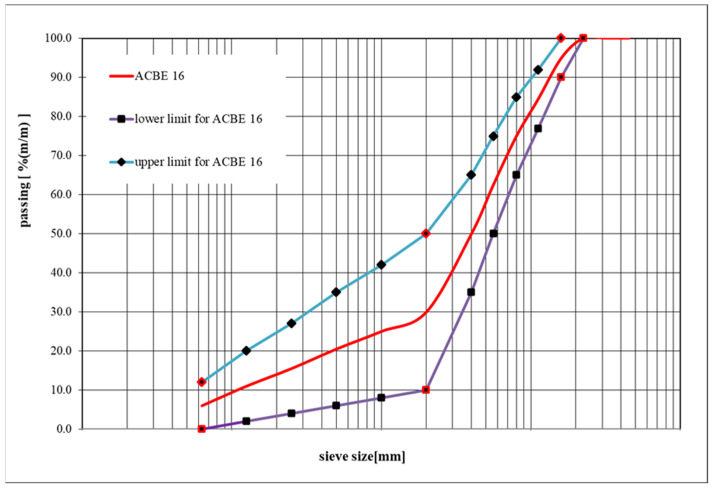
Gradation curve of the ACBE mineral mix.

The design of the mixture of asphalt concrete with bitumen emulsion began with the correct design of the mineral skeleton. Crushed mineral aggregate from a quartzite aggregate mine was used in the mix. Thus, the designed mineral mix [MM] had a grain size consistent with the requirements of [30]. The asphalt concrete mixture is continuously graded. The following figure (Figure 2) shows the grain size curve.

The next stage of the ACBE mix design process involved designing the amount of binders. In line with the concept, the authors provided for the use of four binders in the mix, i.e., bitumen emulsion (A), multicomponent Portland cement (C), synthetic latex (B), and redispersible polymer powder (RPP). As a result of the assumptions, the final composition of the MCE mix was as shown in Table 1.

The composition of the mixture of asphalt concrete with bitumen emulsion (ACBE) was designed in accordance with the guidelines of EN 13108-31 [30] in terms of the limit curves. A grain size of up to 16 mm was adopted. The mineral mix forming the skeleton of ACBE mixtures consisted of quartzite chippings with a grain size of 0/2 at 40% (m/m), 2/5 at 24% (m/m), 5/8 at 12% (m/m), 8/11 at 12% (m/m) and 11/16 at 12% (m/m). Table 2 below shows the properties of the aggregates used in the composition of the mineral mix.

The production of a mixture of asphalt concrete with bitumen emulsion requires an appropriate approach in terms of composition design. An attempt was made to identify the optimum proportion of binders in order to ensure adequate performance of the finished mix. The authors used the following in the project:

- Bitumen emulsion (A);
- Portland cement (C);
- Synthetic latex (B);
- Redispersible polymer powder (P).

ACBE mixtures contain asphalt, which is an ingredient of bitumen emulsion. Bitumen emulsion is added to ACBE mixtures, and when mixed with the mixture components, it breaks down into bitumen and water with additives. Asphalt from the bitumen emulsion coats the aggregate grains, acting as a binder in the mix.

A 70/100 viscosity road bitumen was used to produce the binder. The asphalt emulsion meets the requirements of the national annex to the standard for cationic asphalt emulsions—PN-EN 13808 [38] and is designated C60B10R.

According to the definition of the standard [38], this designates a cationic asphalt emulsion (C) with an emulsion asphalt content of 60% (60), produced from road bitumen (B), with a degradation class of 10 (10), intended for recycling (R).

Table 3 summarises the parameters characterising the asphalt emulsion used.

The mixtures were prepared using fly ash Portland cement of class II, with a strength of 42.5 MPa and with a high early strength “R”, as determined in accordance with EN 197-1 [40]. The main properties of the hydraulic binder used are shown below in Table 4.

Another ingredient used in the project was the modifier for cationic bitumen emulsions—synthetic latex in liquid form. Synthetic latex is a mixture of styrene-butadiene copolymer (styrene-butadiene dispersion) in the amount of 64 (%; m/m) and water together with water-soluble components, e.g., HCl in the amount of 34 (%; m/m). It was dosed into the bitumen emulsion as a binder modifier. Mixtures of asphalt concrete with bitumen emulsion have been made with the addition of a polymer belonging to the plastomer group [44]. The polymer used is a thermoplastic EVA (polyethylene-co-vinyl acetate) copolymer. It comes in the form of a white powder formed by the evaporation of water from the polymer dispersion. It is obtained in the spray-drying process [39]. When mixed with water, this powder forms a dispersion. An important advantage of this product is that it can be mixed with other dry components, such as cement. The mixture thus created produces a cement-polymer binder.

The redispersible polymer powder introduced into the concrete mix forms a dispersion with the liquid phase of the cement grout acting as the dispersed phase. Polymer base: vinyl acetate—ethylene copolymer (EVA/VAE). Protective colloid: polyvinyl alcohol (PVA). Additives: mineral anti-caking agents (calcium carbonate (CaCO_3_)). Due to the start of the hydration process and the loss of water in the mixture, the polymer particles move closer together, forming a closely packed structure and, in the next phase, a continuous layer. A key element in the formation of a continuous polymer phase is coalescence, a phenomenon during which the particles of the dispersed phase are bound together. The phenomenon leads to a reduction in the interfacial surface, which is a very favourable phenomenon [45]. The chemical composition of the redispersible polymer powder is given in Table 5.

According to an analysis of the studies [44,46,47,48], the use of the above-mentioned polymer modifier can improve the mechanical properties of the mixture. This is caused by the occurrence of cross-links between the particles, which produce a continuous polymer phase [44].

The materials described were used to prepare the ACBE mixture with additives. In order to obtain a thoroughly homogeneous mixture, the ingredients were mixed in a WIRTGEN WLM 30 laboratory mixer. Each time, 30 kg of the ingredients was weighed, which was an amount that allowed for thorough mixing.

The specimens made in the laboratory were compacted according to the requirements of the test method [30], i.e., Marshall specimens were compacted statically, using a hydraulic press capable of maintaining constant pressure. The specimens were compacted by applying a load corresponding to 11.9 MPa, maintained continuously for 300 s.

The amount of the material was selected in such a way that the specimen after compaction had a height of 63.5 mm (+/− 2 mm) in accordance with the standard. The specimens prepared this way on the first day after production were stored at room temperature +20 ± 5 °C, on perforated base stands to guarantee uniform drying.

### 2.2. Scope Research and Experiment Design

The scope of research was to verify the effect of binders on the parameters of the ACBE mix. The variables were controlled in the experiment to identify their effect on the properties of the ACBE mixture. The variables included bitumen emulsion (A), Portland cement (C), synthetic latex (styrene–butadiene rubber SBR) (B), and redispersible polymer powder EVA (polyethylene–co-vinyl acetate) (P). ACBE mixtures exhibit characteristics similar to those of cold-recycled deep-cycle mixtures. The authors have experience using redispersible polymer powder and cement [30,31,32,33] in this type of mixture. Additionally, the use of synthetic latex was intended to simulate the use of an emulsion modified with SBR synthetic latex. The analysis of the impact of binders was based on a 34 design modified using the A-optimal optimisation algorithm. In accordance with the experiment design, each of the controlled variables had a different content. Asphalt was added to the ACxBE mix at a rate of 2.00% to 6.00% with an interval of 1.00%. Multicomponent Portland cement was dosed at 0.00% to 2.25% with a 0.75% interval. Synthetic latex was used at a rate of 0.00% to 6.00% with an interval of 2.00%. Redispersible polymer powder was added at a rate of 0.00% to 3.00% with an interval of 1.00%. To assess the properties of the ACBE mix, the authors used well-known test methods commonly used in the testing of mineral-asphalt mixtures. Table 6 shows the scope of laboratory tests.

The initial experiment design used to assess the impact of the modifiers was 34 designs. The design provides for independent variables of a qualitative as well as quantitative type at three levels. Implementing the full design (Response Surface Methodology, denoted as RSM) would require enormous amounts of materials and time. For this task, adopting a second-order response-surface regression model in the form below was needed—Formula (1):

(1) $$Y=\beta_{0}+\sum_{i=1}^{4}\beta_{i}X_{i}+\sum_{i=1}^{4}\beta_{ij}X_{i}X_{j}+\sum_{i<j}\beta_{ij}X_{i}X_{j}$$

where Y is the response variable (Vm, nw, ITS_dry_, ITSR, or Sm), and $X_{1},X_{2},X_{3},X_{4}$ are the coded levels of the four factors: (1) A—asphalt content [%], (2) C—Portland cement content [%], (3) B—synthetic latex content [%], (4) P—redispersible polymer powder content [%].

In this case, the number of combinations would be 81. The initial design was modified using an A-optimal optimisation algorithm. The goal was to construct a new experiment design that would provide for the following:

- excluding cases that were not feasible (extreme cases in the experiment design),
- considering the economic factors (the budget of the research programme),
- minimising the error of the estimation of the future response surface.

The A-optimal design provides many options to select from a list of acceptable points, i.e., design layouts that, for the selected model, would provide the maximum amount of information about the researched area. It is therefore necessary to create a list of proposed points, indicate the model to be fitted to the measurement results and specify the required number of design layouts. The algorithm used allowed for the creation of a new plan consisting of the required number of layouts, with the columns of the design matrix still orthogonal to each other to the maximum possible extent. The rationale for A-optimality is discussed, for example, in Box and Draper [53]. Due to the required calculations, the update of the matrix trace (for the A-optimality criterion) is a slow and computationally demanding process. The A-optimality criterion, characterised by the minimisation of the trace of the inverse of the X’X matrix, was selected for the optimisation process [54]. The maximum value of the following criterion (G-optimality) was used as an optimisation criterion—Formula (2).

(2) $$G_{optimal}=100\times \frac{\sqrt{\frac{p}{N}}}{\Sigma M}$$

where p—number of effects, N—number of required layouts, ΣM—maximum standard deviation of the predicted value of the dependent variable, including all proposed points.

A G-optimal design is defined as one that minimises the largest standard deviation of the determinable response surface. This task was performed using Statistica (version 13.3) software (with the DoE module). After an iterative process taking into account the initial conditions and the objective function of the optimisation process, the authors identified 23 significant cases (reduced from 81 for a full factorial design) to predict the model with the lowest aberration for the assumption of maximising the response satisfying the orthogonality postulate. The optimisation process was closely related to the previously adopted model of the second-degree polynomial, which takes into account the presence of independent factors and the interactions between them. In this case, it is possible to determine a non-linear response surface function in the form of a polynomial of degree two.

The authors have also prepared a reference mix, designed as asphalt concrete with bitumen emulsion, for a binder course with a grain size of up to 16 mm, the same as that specified in the project. The mix contains only 4.75% of asphalt in its composition. The mineral structure of the reference mix was designed in concert with the other mixes used in the project. The asphalt content in the mix was determined using the Duriez Method [55]. This method is based on determining the specific surface area of the aggregate used in the designed mix.

In accordance with the experiment design, each of the controlled variables had a different content. The experimental domain is shown in Table 7 below.

The number of modifiers used has been selected on the basis of literature analysis, own experience, and requirements applicable in Poland [30]. Table 8 delineates the experimental design as a second-order Response Surface Methodology (RSM) employing an A-optimal design alongside regression-based coefficient estimation.

To facilitate the analysis and description of the research work, all mixtures were designated using a code Formula (3). The capital letter of the alphabet (A, C, B, P) indicates the ingredient, while the number indicates the percentage of each ingredient in the mixture. For example, the mixture code reads:

A4C0.75B6P1(3)

where A4—asphalt content in the ACBE mix, at 4.00% (m/m); C0.75—multicomponent Portland cement content in the ACBE mix, at 0.75% (m/m); B6—synthetic latex content in the ACBE mix, at 6.00% (m/m); P1—redispersible polymer powder content in the ACBE mix, at 1% (m/m).

### 2.3. Research Methods

#### 2.3.1. Air Void Content

The first parameter to be analysed is the air void content (Vm) defined by EN 12697-8 [49] as the volume of air voids in the specimen expressed as a percentage of the total volume of the specimen. This dependence is described according to Formula (4).

(4) $$V_{m}=\frac{\rho_{m}-\rho_{b}}{\rho_{m}}\;*\;100\%$$

where V_m_—air void content [0.1%], ρ_m_—density of the mineral-asphalt mix [Mg/m^3^], ρ_b_—bulk density of the mineral-asphalt mix [Mg/m^3^] [56]

#### 2.3.2. Water Absorption by Weight

Another assessed parameter is the water absorption of specimens of asphalt concrete with bitumen emulsion. Water absorption (nw) is the mass and volume of water absorbed by a specimen immersed in water for 24 h at +25 ± 5 °C. After that time, the specimen is removed from the water and dried to constant weight. Water absorption by weight (nw) is calculated as a percentage (m/m) with an accuracy of 0.1% using Formula (5) [57]:

(5) $$n_{w}=\frac{m_{1}-m}{m}*100\%$$

where n_w_—water absorption by weight [0.1%], m_1_—weight of the specimen saturated with water [g], m—weight of the dry specimen [g],

#### 2.3.3. Indirect Tensile Strength ITS_DRY_

As part of the research work, the authors tested the indirect tensile strength (ITSDRY) of the ACBE mixture. The analysis was carried out on Marshall specimens with a diameter of 101.6 ± 0.3 mm and a height of 62.5 ± 2.5 mm. The test was performed at 25 °C ± 2 °C. The test is carried out by placing the specimens between two plates and subjecting them to compression with a constant displacement rate of 50 ± 2 mm/min. The indirect tensile strength ITSdry is calculated according to Formula (6).

(6) $$ITS_{DRY}=\frac{2\cdot P}{\pi \cdot h\cdot D}$$

where P—maximum failure load of the specimen [N], h—height of the specimen [mm], D—diameter of the specimen [mm].

#### 2.3.4. Water and Frost Resistance ITSR

The authors assessed the resistance to climatic conditions. The test concerned the ITSR index, which allows for an assessment of the impact of water and frost. This parameter is a standard factor describing the strength of a mineral-asphalt mixture [58]. Consequently, it was implemented for the mixture of asphalt concrete with bitumen emulsion. Specimens for the study were conditioned over a period of 28 days after compaction. After this time, the specimens provided for testing were subjected to conditioning in water and a freeze–thaw cycle. The test was then carried out at 25 °C ± 1 °C, and the result of the ITSR parameter was calculated using Formula (7).

(7) $$\mathrm{ITSR}=\frac{{\mathrm{ITSR}}_{\mathrm{WET}}}{{\mathrm{ITS}}_{\mathrm{DRY}}}$$

where ITSR—frost and water resistance [%], ITSR_WET_—indirect tensile strength of the conditioned specimens at 25 °C [kPa], ITS_DRY_—indirect tensile strength at 25 °C of unconditioned specimens [kPa].

#### 2.3.5. Stiffness Modulus

The stiffness modulus is an extremely important parameter for mixtures used in road pavement courses. To determine this parameter in the ACBE mixture, a test was performed in the IT-CY indirect tensile scheme. The test was carried out in accordance with PN-EN 12697-26 [52]. The horizontal displacement is 5 ± 2 µm, and the loading time is 124 ± 4 ms.

The stiffness modulus is determined using Formula (8), and the Poisson ratio is determined from Formula (9):

(8) $$S_{m}=\frac{F\cdot (\nu +0.27)}{z\cdot h}$$

(9) $$\nu =3.59\cdot \frac{z}{\Delta V}-0.27$$

where S_m_—stiffness modulus of the specimen [MPa]; F—maximum force applied to the specimen [N]; ν—temperature-dependent Poisson ratio; z—amplitude of horizontal displacement of the specimen under load [mm]; h—thickness of the specimen [mm]; ΔV is the maximum vertical displacement of the specimen (corresponding to the maximum horizontal displacement) [mm].

### 2.4. Sample Replication

The research work was carried out according to the discussed experiment design. The laboratory tests made it possible to determine the physical and mechanical properties, which further made it possible to compare the mixtures of asphalt concrete with bitumen emulsion and indicate the impact of individual modifiers.

In order to obtain a correcC60t analysis of the properties and reliable results, six replications were performed for each ACBE mixture test. Each time, the experiment results were evaluated within a confidence interval with an assumed probability (*P* = 95%). This method of analysis made it possible to identify measurements with an error and eliminate them from further consideration. The requisite number of repetitions was determined by the necessity to stabilise the variance within the experimental design, which had been modified by the A-optimal algorithm, in conjunction with standard methodological requirements. The confidence interval was calculated according to Formula (10):

(10) $$\bar{x}\pm 1.96(\frac{\sigma }{\sqrt{n}})$$

where $\bar{x}$—expected value, 1.96—statistic for significance level α = 0.05, σ—standard deviation, n—sample size.

Planning an experiment is an extremely complex task. The common method of testing “one variable at a time” requires a significant amount of time and financial resources [59,60]. The overriding aim of planning an experiment is to obtain an answer to the question posed in an efficient manner, which means that the selection of an appropriate experiment design becomes a crucial task. Choosing the correct plan at the very beginning saves plenty of time and also money.

## 3. Research Results and Optimisation

### 3.1. Analysis of Research Results

In accordance with the adopted research design, the authors assessed the influence of modifiers on the properties of the mixture of asphalt concrete with bitumen emulsion (ACxBE). The assessment was carried out in accordance with the adopted research design, which made it possible to build mathematical models describing the analysed characteristics.

**Figure 3:**
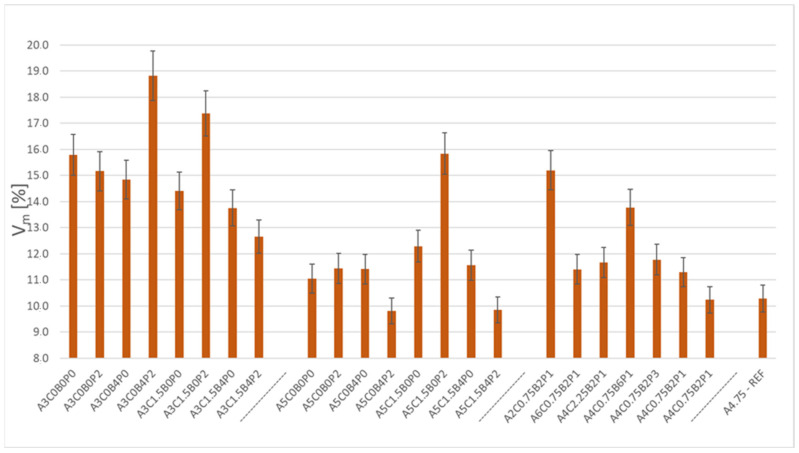
Air void content V_m_ in the analysed mixtures.

The first analysed parameter was the air void content (Vm). The obtained research results are shown in Figure 3. In the diagram, the mixtures are ranked according to the experiment design. The air void content (Vm) of the analysed mixtures of asphalt concrete with bitumen emulsion ranges between 9.8% and 18.8%.

It is worth noting that the use of modifiers has a significant impact on the value of the analysed parameter. This is observable in the case of the A5C1.5B4P0 mix, which has an air void content of 11.6%. After adding 2% of redispersible polymer powder, the air void content is reduced. The mix became more workable, resulting in an air void content of 9.9%. What is more, the addition of cement also has a beneficial effect on reducing the air void content. The A3C0B0P0 mix has an air void content of 15.8%, and with the addition of 1.5% of cement (A3C1.5B0P0 mix), the air void content decreases by approximately 1.5% to 14.4%. The average air void content of the mixtures is 13.1%. The A5C0B4P2 mix has the lowest air void content, and its Vm is 9.8%. For the mixture with the highest air void content, A3C0B4P2, this parameter is 18.8%. An air void content of 10.3% was recorded for the A4.75-REF reference mix. The effect of the analysed factors on the considered Vm parameter in the ACBE mixture is shown in Table 9.

Table 9 (as well as Table 10, Table 11, Table 12 and Table 13) presents the results of a response-surface regression analysis. For each property (V_m_, n_w_, ITS_dry_, ITSR, S_m_), a second-order polynomial model was fitted in terms of the coded contents of bitumen (A), cement (C), synthetic latex (B), and redispersible polymer powder (P). The tables list the regression coefficients (“Factor”), their t-statistics and *p*-values, together with the intercept, the coefficient of determination R^2^, and the mean square of pure error (Pure error MS). Linear [(L)], quadratic [(Q)] and two-factor interaction terms (e.g., 1L × 2L) are reported. In table notation, “1L×2L” means interaction between the linear effect of factor 1 (A) and the linear effect of factor 2 (C). Finally, it describes the interaction between bitumen and cement additive.

**Figure 4:**
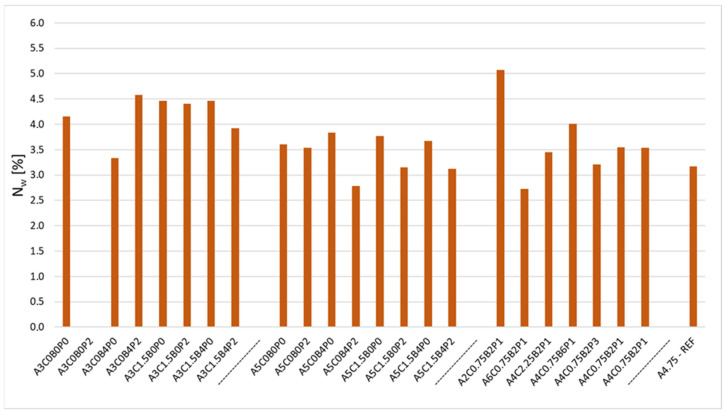
Air void content n_w_ in the analysed mixtures.

Another analysed parameter is water absorption (N_w_). Water absorption by weight is an important parameter of the mix due to its position in the system of road pavement courses. The mixtures used in road construction are constantly exposed to moisture as a result of, for example, rainfall or capillary rise. The results for the experimental ACBE mixtures are shown in the chart (Figure 4).

The analysed mixtures have a water absorption ranging from 2.7% to 5.1%. The reference mix had a water absorption of 3.2%. The lowest water absorption value was recorded for the A6C0.75B2P1 mixture. This is due to the highest asphalt content in the mix, which tightly seals the structure of the mixture. In comparison, the A2C0.75B2P1 mix with the lowest asphalt content has the highest water absorption of those analysed—5.1%. During the research, the authors encountered a problem with the A3C0B0P2 mixture, whose specimens did not survive the period of conditioning in water. The mixed specimens were destroyed, and it was not possible to determine their water absorption by weight. The authors also observed a beneficial effect of the combination of polymer powder and synthetic latex. Every time the two ingredients were used, this had a positive impact on reducing water absorption. Table 10 below shows the impact of the analysed factors on the n_w_ parameter.

**Figure 5:**
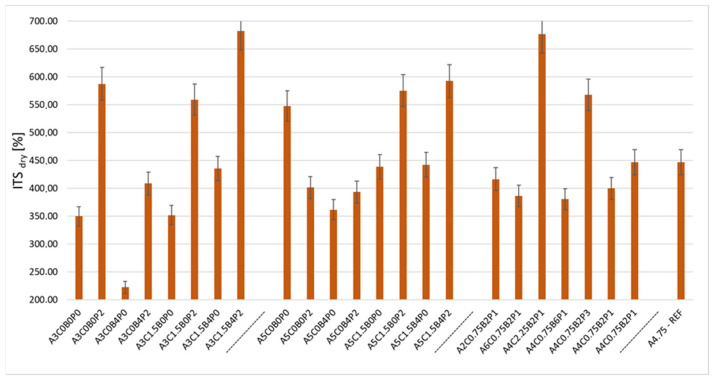
Indirect tensile strength ITSdry of ACBE mixtures.

The next analysed parameter is the indirect tensile strength ITS_DRY_. The test results for the mixtures of asphalt concrete with bitumen emulsion ranged from 222.26 kPa to 682.22 kPa. The results of the indirect tensile strength obtained from the ACBE mixtures tested in the experiment are shown in the chart (Figure 5).

The results of the ITS_DRY_ tests above show a large variation in the obtained results. This is due to the varied composition of the mixes. The achieved ITS_DRY_ value is significantly affected by the content of cement and redispersible polymer powder in the mix. This can be seen in the A5C1.5B0P2 mixtures. This mixture had an ITS_DRY_ of 575.23 kPa. In comparison, the corresponding mixture A5C1.5B0P0 had an indirect tensile strength of 438.48 kPa. The significant influence of the polymer powder is also evident in the A4C0.75B2P3 mixture. An ITS_DRY_ of 567.78 kPa was recorded for the mixture. The mixture with a reduced share of modifier P, A4C.07B2P1, had a value of 446.85 kPa. A 2% decrease in the polymer share resulted in a decrease in ITS_DRY_ by approximately 20%. The phenomenon is caused by polymer cross-linking and hydration of the cement in the mix. These mechanisms result in the stiffening of the mix. The negative impact of using synthetic latex is also noticeable. A mixture containing only 3% asphalt, designated A3C0B0P0, had an ITS_DRY_ of 349.70 kPa. The addition of 4% of latex reduced this value to 222.26 kPa. A similar phenomenon can be observed for the A5C0B0P0 mix, which recorded 547.69 kPa. Adding 4% latex to this mixture also had a negative effect, reducing the indirect tensile strength. The mixture, designated A5C0B4P0, recorded 361.63 kPa. The highest indirect tensile strength was recorded for the A3C1.5B4P2 mixture. It had an ITS_DRY_ of 682.22 kPa. The lowest value of the parameter under consideration was obtained for the A3C0B4P0 mixture—222.26 kPa. The indirect tensile strength for the A4.75-REF mix was 446.65 kPa. The observed decrease in indirect tensile strength (ITS) is caused by the lower adhesion of the asphalt precipitated from the emulsion to the aggregate grains. Adding latex to the asphalt emulsion at ambient temperatures, around 20 °C, changes the emulsion’s viscosity, causing it to increase, which hinders uniform coating and inter-grain bonding. Furthermore, adding latex to the asphalt emulsion changes the surface tension and reduces the adhesion of the asphalt precipitated from the emulsion to the aggregate grains. This phenomenon is exacerbated by the fact that the ingredients are mixed “cold,“ at around 20 °C.

The effect of the analysed factors on the considered ITS_DRY_ parameter in the ACBE mixture is shown in Table 11.

**Figure 6:**
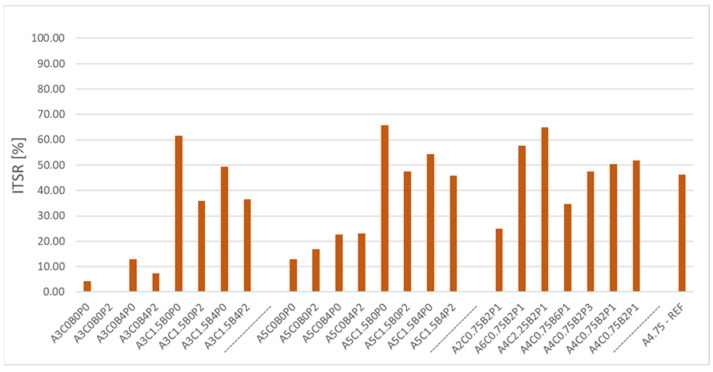
ITSR water and frost resistance of ACBE mixtures.

From the perspective of the durability of ACBE mixtures, it is important to analyse frost and water damage resistance. Figure 6 below shows the results obtained from measurements of the harmful effects of water and frost on the mixture of asphalt concrete with bitumen emulsion.

The chart above shows the recorded values for ITSR water and frost damage resistance in indirect tension. Of those analysed, only three mixtures achieved an ITSR of more than 60%. These mixtures are A3C1.5B0P0, A5C1.5B0P0, and A4C2.25B2P1. Each of the mixtures contained cement at a rate of more than 1.5%. The other modifiers made up a smaller proportion of the mix. It is therefore apparent that the use of cement has a significant effect on the parameter under analysis. In addition, this effect can be observed by comparing the mixtures containing only asphalt, i.e., A3C0B0P0 and A5C0B0P0, and juxtaposing them with corresponding mixes containing 1.5% of cement, i.e., A3C1.5B0P0 and A5C1.5B0P0. In each case, the mixtures with cement recorded values higher than 60%. A similar result was achieved by the A6C0.75B2P1 mixture, with an ITSR of 57.56%. This was significantly affected by the asphalt content of the mix, which prevented the propagation of water into the mix structure. This is apparent in the corresponding mixture containing 4% less asphalt—A2C0.75B2P1. This mixture recorded 24.90% resistance. Furthermore, the authors also recorded the negative effects of synthetic latex on ITSR. This is manifested in A4C0.75B6P1 and A4C0.75B2P1 mixes. The mixture containing 6% of synthetic latex has a water and frost damage resistance of 34.76%. Reducing the modifier content increased ITSR to 51.79%. A water and frost resistance of 46.23% was recorded for the reference mix referred to as A4.75-REF. It should also be noted that during the research, one of the mixtures did not survive until the tests. During the cycle of soaking in water, this mixture disintegrated. This indicates zero frost and water damage resistance. The worst test results were recorded for mixtures with 3% asphalt and zero cement content. These mixtures had resistance from 0.00% to 12.95%. In each case, the cement content had a positive effect on the recorded ITSR results. The effect of the analysed factors on the considered ITSR parameter in the ACBE mixture is shown in Table 12.

**Figure 7:**
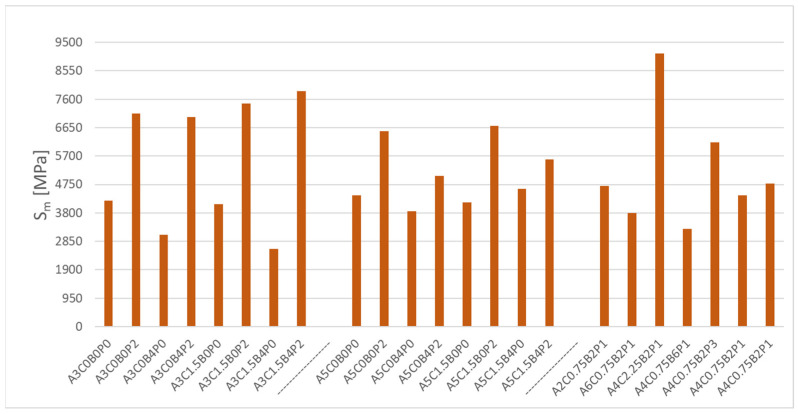
Value of the stiffness modulus S_m_ tested using the IT-CY method for ACBE mixtures.

The last analysed parameter is the stiffness modulus of asphalt concrete with bitumen emulsion. Figure 7 below shows the results obtained from measurements. The test was conducted at 13 °C.

The chart above (Figure 7) accurately illustrates the variation in the stiffness modulus depending on the types and amounts of binder used. There is a noticeable effect of the use of polymer powder on the increase in the value of the analysed parameter. The A3C0B0P2 mixture had a modulus of 7130 MPa. The lack of polymer in the A3C0B0P0 mix resulted in a stiffness modulus of 4202 MPa. Similarly, for the A5C0B0P0 mix containing 5% asphalt. The mixture achieved a stiffness modulus of 4381 MPa. With the addition of 2% of polymer powder, the stiffness modulus increased to 6533 MPa. There is also a significant impact of synthetic latex on the analysed parameter. The use of this ingredient leads to a decrease in the stiffness modulus. This is noticeable for A4C0.75B6P1 and A4C0.75B2P1 mixtures. The former of the mixtures achieved a stiffness modulus of 3275 MPa in the tests, whereas the reduction in latex share by 4% increased the modulus to 4778 MPa. Cement also played an important role. Increasing its content in the mixes leads to an increase in the stiffness modulus, as can be seen in the A4C2.25B2P1 mix, which had a stiffness modulus of 9128 MPa—the highest recorded modulus in the entire group of tested mixtures. No stiffness modulus test was carried out for the reference mix, which is why it does not appear in the chart. The effect of the analysed factors on the considered Sm parameter in the ACBE mixture is shown in Table 13.

### 3.2. Optimisation

The optimisation process involves adopting criteria whose change leads to significant changes in the context of the properties of the mixture. Accordingly, a multi-criteria optimisation of the mixture composition was carried out in order to proceed to the analyses in the second phase of the research. This method uses a general utility function [61,62] and is characterised by expression on a dimensionless scale. This type of scale requires the imposition of a range of satisfactory values. The individual criteria are described by non-negative coefficients reflecting their validity for a given mixture. The sum of the coefficients must be 1. A researcher carrying out an optimisation of this kind must be familiar with the technical requirements of the phenomenon. Using the described methodology, the authors determined the optimum quantities of the ingredients of the mixture of asphalt concrete with bitumen emulsion analysed in the project, i.e., asphalt, cement, synthetic latex, and redispersible polymer powder. The general utility function U_i_^III^ contains numbers from the range (0;1). Table 14 below shows the qualitative intervals of the function.

The utilities attributed to the individual features y(i) are determined by two algorithms, and their profiles are shown in [49]. The general utility UIII, also denoted as D, is assumed to be the weighted geometric mean of the individual values of Formula (11):

(11) D=∏u=1ndu1n

where *n*—number of variables

Utility function profiles illustrate two possible cases that can be encountered in the optimisation process. The optimisation task is to search for a solution range or a specific solution where the utility function adopts a value of 0.37 or higher. The optimal solution, therefore, is a result obtained in the optimisation process, taking into account predetermined criteria. Every change in the criteria significantly affects the result of the estimation of the desired outcome in terms of product properties. The work involved the optimisation of the mixture of asphalt concrete with bitumen emulsion in terms of the percentage share of asphalt, cement, synthetic latex, and redispersible polymer powder. Optimisation was carried out using the criteria shown in Table 15 below.

The optimisation process used the models shown in Table 16. In the absence of existing guidelines for the design of the mixtures of asphalt concrete with bitumen emulsion, it was decided to use the guidelines for the design of mineral-asphalt mixtures (WT2—2016) [58] and the guidelines for the design of MCE mixtures [63]. Both documents are binding regulatory documents for the design and preparation of mixes in Poland. Table 16 below shows the material models for ACBE mixes.

The optimisation resulted in eight mixtures that best match the optimisation criteria. The composition of the mixtures is included in Table 17 below.

**Figure 8:**
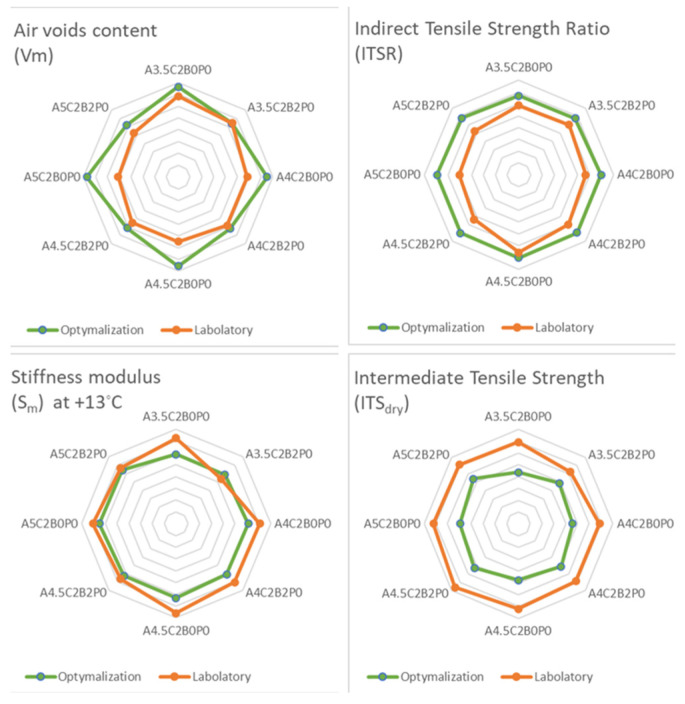
Fit of optimisation results.

A check was performed to verify the predicted models in practice. The specimens were prepared according to the predicted mixture type, and then tests under laboratory conditions were performed. The authors carried out the tests specified in the optimisation criteria. Figure 8 below shows the fit between the laboratory test results and the design parameters of the optimisation.

The obtained research results indicate great potential and the possibility of using this technology as an alternative to HMA. However, it should be emphasised that the use of the ACxPB mixture should be limited to the lower layers of the structure. This requires a detailed analysis of the effect of modifiers on rheological properties and fatigue life. Recommendations for typical structural layers are also necessary after identifying the material constants used in mechanistic structural design methods. The analysed mixture offers significant environmental benefits. Production takes place at ambient temperature, without heating the aggregate. This reduces the harmful effects of environmental pollution. It also leads to financial savings by reducing the fuel consumption needed to heat the aggregate. Furthermore, the mixture can be placed without a persistent odour, which is negatively perceived by society.

## 4. Conclusions

The test results and analyses of the impact of the binders on the properties of ACBE asphalt concrete with bitumen emulsion support the following conclusions:

- The influence of synthetic latex is evident in the composition of the ACBE mixture. Mixtures containing modifier B are characterised by better workability, with a resultant reduction in air void content.
- The use of 1.5% cement in the mix composition has a beneficial effect on the stiffness modulus and indirect tensile strength. The use of a small amount of hydraulic binder may be necessary at the early stages of pavement construction using the ACBE mixture.
- Redispersible polymer powder adversely affects the ITSR of the mix. Specimens containing the modifier P have low frost and water damage resistance.
- The use of low asphalt content (less than 4%) and polymer powder in the mix leads to a weaker structure, compromising frost and water damage resistance.
- The use of redispersible polymer powder (P) significantly improves the workability of the mixture, as can be seen when analysing the air void content Vm.
- On the basis of the optimisation, it should be concluded that the best parameters are achieved by the C4.5C2B0P0 mixture. The other mixtures have low frost and water damage resistance.
- Rheological and fatigue life studies should be considered as future research directions.

## Figures and Tables

**Table 1:** Percentage of mineral mix ingredients.

Ingredient	Origin	Density [g/cm^3^]	MM [%, m/m]
0/2 fine aggregate	Quartzite	2.68	35.00–38.80
2/5 quartzite chippings	Quartzite	2.63	21.00–23.30
5/8 quartzite chippings	Quartzite	2.66	10.50–11.60
8/11 quartzite chippings	Quartzite	2.66	10.50–11.60
11/16 quartzite chippings	Quartzite	2.65	10.50–11.60
Bitumen emulsion	-	1.00	3.33–10.00
Multicomponent Portland cement	-	2.99	0.00–2.25
Synthetic latex	-	1.00	0.00–6.00
Redispersible polymer powder	-	0.50	0.00–3.00

**Table 2:** Properties of the aggregates used in the project.

Property	Test	Unit of Measure	Symbol				
**Size**	EN 933-1 [31]	-	0/2	2/5.6	5.6/8	8/11	11/16
**Grain size**	EN 933-1 [31]	-	G_F_85	Gc85/20	Gc85/20	Gc90/15	Gc85/35
**Density**	EN 1097-6 [32]	Mg/m^3^	2.68	2.63	2.66	2.66	2.65
**Flakiness index**	EN 933-3 [33]	%	-	Fl_20_	Fl_15_	Fl_15_	Fl_10_
**Percentage of crushed or broken grains**	EN 933-5 [34]	%	-	C_100/0_	C_100/0_	C_100/0_	C_100/0_
**Frost resistance**	EN 1367-1 [35]	%	-	F_1_	F_1_	F_1_	F_1_
**Crushing resistance of coarse aggregate**	EN 1097-2 [36]	%	-	LA_25_	LA_25_	LA_25_	LA1_5_
**Resistance to abrasion**	EN 1097-1 [37]	%	-	M_DE_10	M_DE_10	M_DE_10	M_DE_10

**Table 3:** Properties of the C60B10 R bitumen emulsion [39].

Property	Test	Unit of Measure	Value
Binder content	PN-EN 13808 [38]	% (m/m)	60.0
Stability of mixing with cement		g	0.3
Percent retained on 0.5 mm sieve		% (m/m)	0.06
Ø2 mm efflux time at 40 °C		s	27
Asphalt-aggregate bond strength		%	75
Reclaimed asphalt penetration grade		0.1 mm	53
Softening point of reclaimed asphalt		°C	55.2

The amount of emulsifier in the emulsion composition was 1.2%. The size of dispersed asphalt particles ranged from 1 to 20 microns, with the largest share being particles of 4 microns.

**Table 4:** Properties of CEM II 42.5R Portland cement.

Property	Test	Unit of Measure	Value
Initial setting time	EN 196-3 [41]	min	246
Compressive strength at 2 days	EN 196-1 [42]	MPa	26.2
Compressive strength at 28 days	EN 196-1 [42]	MPa	57.5
Soundness	EN 196-3 [41]	mm	0.8
Specific surface area	EN 196-6 [43]	cm2/g	4354

**Table 5:** Chemical composition of the EVA polymer [46].

Component	Participation [%]
C	67.67
O	29.13
Mg	0.52
Si	1.65
Ca	0.75
Al	0.29

**Table 6:** Scope of research.

Property	Test Standard
Air void content (V_m_)	PN-EN 12697-8 [49]
Water absorption by weight (n_w_)	PN-S 04001-12 [50]
Indirect tensile strength (ITS)	PN-EN 12697-23 [51]
Stiffness modulus S_m_ at 13 °C	PN-EN 12697-26 [52]

**Table 7:** Experiment domain.

	MIN [%]	MAX [%]	STEP [%]
Asphalt (A)	2.00	6.00	2.00
Portland Cement (C)	0.00	2.25	0.75
Synthetic Latex (B)	0.00	6.00	2.00
Pedispersible polymer powder (P)	0.00	3.00	1.00

**Table 8:** Experiment design for the evaluation of the impact of redispersible polymer powder on the properties of the MCE mix using RSM employing an A-optimal design alongside regression-based coefficient estimation.

Type of Mix ACBE	A (Asphalt) [%]	C (Portland Cement) [%]	B (Synthetic Latex) [%]	P (Redispersible Polymer Powder) [%]
A3C0B0P0	3.00	0.00	0.00	0.00
A3C0B0P2	3.00	0.00	0.00	2.00
A3C0B4P0	3.00	0.00	4.00	0.00
A3C0B4P2	3.00	0.00	4.00	2.00
A3C1.5B0P0	3.00	1.50	0.00	0.00
A3C1.5B0P2	3.00	1.50	0.00	2.00
A3C1.5B4P0	3.00	1.50	4.00	0.00
A3C1.5B4P2	3.00	1.50	4.00	2.00
A5C0B0P0	5.00	0.00	0.00	0.00
A5C0B0P2	5.00	0.00	0.00	2.00
A5C0B4P0	5.00	0.00	4.00	0.00
A5C0B4P2	5.00	0.00	4.00	2.00
A5C1.5B0P0	5.00	1.50	0.00	0.00
A5C1.5B0P2	5.00	1.50	0.00	2.00
A5C1.5B4P0	5.00	1.50	4.00	0.00
A5C1.5B4P2	5.00	1.50	4.00	2.00
A2C0.75B2P1	2.00	0.75	2.00	1.00
A6C0.75B2P1	6.00	0.75	2.00	1.00
A4C2.25B2P1	4.00	2.25	2.00	1.00
A4C0.75B6P1	4.00	0.75	6.00	1.00
A4C0.75B2P3	4.00	0.75	2.00	3.00
A4C0.75B2P1	4.00	0.75	2.00	1.00
A4C0.75B2P1	4.00	0.75	2.00	1.00
A4.75-REF	4.75	0.00	0.00	0.00

**Table 9:** Effect of the analysed factors using RSM on the considered V_m_ parameter.

	V_m_; R^2^ = 0.81; Pure Error; MS = 0.6058		
	*t*	Factor	*p* ≤ 0.05
Intercept	16.26	27.90	<0.0001
(1)A[%](L)	−7.44	−6.21	<0.0001
A[%](Q)	4.92	0.50	<0.0001
(2)C[%](L)	−2.39	−1.61	0.020
C[%](Q)	−1.23	−0.27	0.224
(3)B[%](L)	3.86	0.97	<0.0001
B[%](Q)	5.32	0.16	<0.0001
(4)P[%](L)	−2.19	−1.10	0.032
P[%](Q)	2.73	0.34	0.008
1L × 2L	8.04	1.03	<0.0001
1L × 3L	−4.53	−0.22	<0.0001
1L × 4L	1.03	0.10	0.307
2L × 3L	−13.82	−0.88	<0.0001
2L × 4L	4.03	0.51	<0.0001
3L × 4L	−6.03	−0.29	<0.0001

“R^2^”—coefficient of determination; “MS”—mean square of the pure experimental error; “Intercept”—intercept of the regression model; Factor—estimated regression coefficient; “*t*”—*t*-statistic for testing H_0_: β = 0; “*p* ≤ 0.05”—*p*-value of the *t*-test.

**Table 10:** Impact of the analysed factors using RSM on the n_w_ parameter.

	n_w_; R^2^ = 0.77; Pure Error; MS = 0.09106		
	*t*	Factor	*p* ≤ 0.05
Intercept	14.02	9.33	0.000
(1)A[%](L)	−5.63	−1.82	0.000
A[%](Q)	3.08	0.12	0.003
(2)C[%](L)	−9.12	−2.38	<0.0001
C[%](Q)	4.00	0.34	<0.0001
(3)B[%](L)	−14.41	−1.41	<0.0001
B[%](Q)	8.56	0.10	<0.0001
(4)P[%](L)	18.42	3.61	<0.0001
P[%](Q)	−3.14	−0.15	0.003
1L × 2L	8.14	0.41	<0.0001
1L × 3L	10.36	0.19	<0.0001
1L × 4L	−14.49	−0.54	<0.0001
2L × 3L	9.94	0.25	<0.0001
2L × 4L	−13.44	−0.66	<0.0001
3L × 4L	−10.52	−0.20	<0.0001

“R^2^”—coefficient of determination; “MS”—mean square of the pure experimental error; “Intercept”—intercept of the regression model; Factor—estimated regression coefficient; “*t*”—*t*-statistic for testing H_0_: β = 0; “*p* ≤ 0.05”—*p*-value of the *t*-test.

**Table 11:** Impact of the analysed factors using RSM on the ITSdry parameter.

	ITS_dry_; R^2^ = 0.87; Pure Error; MS = 905		
	*t*	Factor	*p* ≤ 0.05
Intercept	2.24	148.59	0.003
(1)A[%](L)	2.80	90.49	0.007
A[%](Q)	−1.13	−4.46	0.264
(2)C[%](L)	−3.82	−99.36	<0.0001
C[%](Q)	8.15	69.30	<0.0001
(3)B[%](L)	−2.88	−28.07	0.005
B[%](Q)	−0.30	−0.36	0.762
(4)P[%](L)	10.32	201.39	<0.0001
P[%](Q)	0.71	3.38	0.482
1L × 2L	−1.48	−7.36	0.143
1L × 3L	−0.75	−1.41	0.453
1L × 4L	−11.72	−43.68	<0.0001
2L × 3L	12.07	29.76	<0.0001
2L × 4L	6.73	33.21	<0.0001
3L × 4L	2.47	4.57	0.016

“R^2^”—coefficient of determination; “MS”—mean square of the pure experimental error; “Intercept”—intercept of the regression model; Factor—estimated regression coefficient; “*t*”—*t*-statistic for testing H_0_: β = 0; “*p* ≤ 0.05”—*p*-value of the *t*-test.

**Table 12:** Impact of the analysed factors using RSM on the ITSR parameter.

	ITSR; R^2^ = 0.90; Pure Error; MS = 1.024		
	*t*	Factor	*p* ≤ 0.05
Intercept	−18.47	−77.29	0.034
(1)A[%](L)	20.04	40.23	0.032
A[%](Q)	−15.73	−3.87	0.040
(2)C[%](L)	36.45	63.04	0.017
C[%](Q)	−25.05	−13.57	0.025
(3)B[%](L)	12.77	8.28	0.050
B[%](Q)	−20.15	−1.54	0.032
(4)P[%](L)	−4.81	−6.24	0.130
P[%](Q)	−1.74	−0.53	0.332
1L × 2L	−8.94	−2.98	0.070
1L × 3L	−4.14	−0.52	0.151
1L × 4L	2.74	0.68	0.223
2L × 3L	−9.45	−1.56	0.067
2L × 4L	−10.16	−3.36	0.062
3L × 4L	9.37	1.16	0.068

“R^2^”—coefficient of determination; “MS”—mean square of the pure experimental error; “Intercept”—intercept of the regression model; Factor—estimated regression coefficient; “*t*”—*t*-statistic for testing H_0_: β = 0; “*p* ≤ 0.05”—*p*-value of the *t*-test.

**Table 13:** Impact of the analysed factors using RSM on the S_m_ parameter.

	S_m_; R^2^ = 0.89; Pure Error; MS = 307,833.4		
	*t*	Factor	*p* ≤ 0.05
Intercept	1.56	1899.87	0.124
(1)A[%](L)	1.52	883.42	0.132
A[%](Q)	−0.95	−66.35	0.346
(2)C[%](L)	−6.49	−3257.42	<0.0001
C[%](Q)	13.14	1984.82	<0.0001
(3)B[%](L)	−0.18	−34.46	0.855
B[%](Q)	−1.49	−31.47	0.140
(4)P[%](L)	10.36	3918.66	<0.0001
P[%](Q)	−3.01	−254.34	0.004
1L × 2L	0.40	39.58	0.687
1L × 3L	−0.41	−14.95	0.684
1L × 4L	−7.31	−535.65	<0.0001
2L × 3L	1.43	69.98	0.158
2L × 4L	1.35	131.71	0.182
3L × 4L	0.08	2.97	0.936

“R^2^”—coefficient of determination; “MS”—mean square of the pure experimental error; “Intercept”—intercept of the regression model; Factor—estimated regression coefficient; “*t*”—*t*-statistic for testing H_0_: β = 0; “*p* ≤ 0.05”—*p*-value of the *t*-test.

**Table 14:** Assessment of the qualitative utility function [61].

Qualitative Range	Interpretation
1.00	Excellent value.
1.00–0.83	A very good value representing the achievement of material quality with extraordinary optimisation properties.
0.8–0.63	This range represents good, above-average quality.
0.63–0.37	Satisfactory (sufficient) value, acceptable under certain conditions.
0.37–0.2	Unacceptable value that may increase the unreliability of the optimised product.

**Table 15:** Optimisation criteria adopted in the project.

	Air Voids Content (V_m_)	Water and Cold Resistance (ITSR)	Stiffness (S_m_), at a Tem. of 13 °C	Intermediate Tensile Strength (ITS_dry_)
	[%]	[%]	[MPa]	[kPa]
Better	1	60	5001	451
Worse	12	59	5000	450

**Table 16:** Material models for ACBE mixes.

Material Models	R^2^
$V_{m}=27.90-6.21\cdot A+0.50\cdot A^{2}-1.61\cdot C-0.27{\cdot C}^{2}+0.97\cdot B+0.16{\cdot B}^{2}-1.10\cdot P+0.34{\cdot P}^{2}+0.34\cdot A\cdot C-0.22\cdot A\cdot B+0.10\cdot A\cdot P-0.88\cdot C\cdot B+0.51\cdot C\cdot P-0.29\cdot B\cdot P$	0.81
${ITS}_{dry}=148.59-90.49\cdot A-4.46\cdot A^{2}-99.36\cdot C+69.30{\cdot C}^{2}-28.07\cdot B-0.36{\cdot B}^{2}+201.39\cdot P+3.38{\cdot P}^{2}-7.36\cdot A\cdot C-1.41\cdot A\cdot B-43.68\cdot A\cdot P+29.76\cdot C\cdot B+33.21\cdot C\cdot P+4.57\cdot B\cdot P$	0.87
$ITSR=-77.29+40.23\cdot A-3.87\cdot A^{2}+63.04\cdot C-13.57{\cdot C}^{2}+8.28\cdot B-1.54{\cdot B}^{2}-6.24\cdot P-0.53{\cdot P}^{2}-2.98\cdot A\cdot C-0.52\cdot A\cdot B+0.68\cdot A\cdot P-1.56\cdot C\cdot B-3.36\cdot C\cdot P+1.16\cdot B\cdot P$	0.90
$S_{m}=1899.87+883.42\cdot A-66.35\cdot A^{2}-3257.42\cdot C+1984.82{\cdot C}^{2}-34.46\cdot B-31.47{\cdot B}^{2}+3918.66\cdot P-254.34{\cdot P}^{2}+39.58\cdot A\cdot C-14.95\cdot A\cdot B-535.65\cdot A\cdot P+69.98\cdot C\cdot B+131.71\cdot C\cdot P+2.97\cdot B\cdot P$	0.89

**Table 17:** Composition of optimal ACBE mixtures.

Number	Symbol	A [%]	C [%]	B [%]	P [%]
1	A3.5C2B0P0	3.5	2.0	0.0	0.0
2	A3.5C2B2P0	3.5	2.0	2.0	0.0
3	A4C2B0P0	4.0	2.0	0.0	0.0
4	A4C2B2P0	4.0	2.0	2.0	0.0
5	A4.5C2B0P0	4.5	2.0	0.0	0.0
6	A4.5C2B2P0	4.5	2.0	2.0	0.0
7	A5C2B0P0	5.0	2.0	0.0	0.0
8	A5C2B2P0	5.0	2.0	2.0	0.0

## Data Availability

The original contributions presented in this study are included in the article. Further inquiries can be directed to the corresponding author.
